# Evidence of local short-distance spawning migration of tropical freshwater eels, and implications for the evolution of freshwater eel migration

**DOI:** 10.1002/ece3.1245

**Published:** 2014-09-16

**Authors:** Takaomi Arai

**Affiliations:** 1 Institute of Oceanography and Environment, Universiti Malaysia TerengganuKuala Terengganu, Terengganu, 21030, Malaysia

**Keywords:** Ancestral form, *Anguilla*, catadromy, maturation, migration

## Abstract

Freshwater eels have fascinated biologists for centuries due to the spectacular long-distance migrations between the eels’ freshwater habitats and their spawning areas far out in the ocean and the mysteries of their ecology. The spawning areas of Atlantic eels and Japanese eel were located far offshore in the Atlantic Ocean and the Pacific Ocean, respectively, and their reproduction took place thousands of kilometers away from their growth habitats. Phylogenetic studies have revealed that freshwater eels originated in the Indonesian region. However, remarkably little is known about the life histories of tropical freshwater eels despite the fact that tropical eels are key to understanding the nature of primitive forms of catadromous migration. This study found spawning-condition tropical freshwater eels in Lake Poso, central Sulawesi, Indonesia, with considerably high gonadosomatic index values and with histologically fully developed gonads. This study provides the first evidence that under certain conditions, freshwater eels have conditions that are immediately able to spawn even in river downstream. The results suggest that, in contrast to the migrations made by the Atlantic and Japanese eels, freshwater eels originally migrated only short distances of <100 kilometers to local spawning areas adjacent to their freshwater growth habitats. Ancestral eels most likely underwent a catadromous migration from local short-distance movements in tropical coastal waters to the long-distance migrations characteristic of present-day temperate eels, which has been well established as occurring in subtropical gyres in both hemispheres.

## Introduction

Schmidt (1922) discovered that the spawning area for both the European eel *Anguilla anguilla* and the American eel *A. rostrata* was located far offshore in the Sargasso Sea of the Atlantic Ocean thousands of kilometers away from their growth habitats in Europe and North America, indicating that these two species of Atlantic freshwater eels make remarkably long-spawning migrations. More recently, the spawning area of the Japanese eel, *A. japonica*, was discovered far offshore in the Philippine Sea of the western North Pacific (Tsukamoto 1992) where all of the oceanic stages of this species were first collected, including spawning adults, eggs, and recently hatched larvae (Tsukamoto et al. 2011). These eels in both the Atlantic and Pacific spawn in similar westward flowing currents at the southern edges of the subtropical gyres in both oceans; therefore, their larvae (leptocephali) can be passively transported to coastal areas. The long migrations to these spawning areas have fascinated scientists because each eel must migrate thousands of kilometers back to the same area for spawning. These discoveries of the spawning areas of temperate eels have stimulated numerous studies regarding the life history and freshwater ecology of these eels, indicating that temperate eels have well-defined spawning seasons.

Nineteen species of freshwater eels have been reported worldwide, 13 from tropical regions. Of the latter, seven species occur in the western Pacific around Indonesia (Ege 1939; Castle and Williamson 1974; Arai et al. 1999). Molecular phylogenetic researches on freshwater eels has recently revealed that tropical eels are the most basal species originating in the Indonesian region and that freshwater eels radiated out from the tropics to colonize the temperate regions (Minegishi et al. 2005). Tropical freshwater eels must be more closely related to the ancestral form than are their temperate counterparts. Thus, studying the biological aspects of tropical eels provide clues for understanding the nature of primitive forms of catadromous migration in freshwater eels and how the large-scale migration of temperate species became established. The recent drastic decline of glass eel recruitment in Europe and East Asia has caused serious problems in eel stock to sustainable levels of adult abundance (Arai 2014). Tropical eels are considered a major target species for compensating for the high demand of eel resources. However, remarkably little is known about the spawning area, spawning ecology, and life histories of the many tropical eels across the Indo-Pacific region.

Until recently, remarkably little was known about the spawning areas of tropical eels. Early in the last century, the Danish Round the World Expedition from 1928 to 1930 collected many leptocephali off west Sumatra of Indonesia, indicating that *Anguilla bicolor bicolor* spawned there near the Mentawai Trench (Jespersen 1942). More recently, *A. celebesensis* and *A. borneensis* were thought to spawn in the Celebes Sea, the Tomini Bay, and the Celebes Sea, respectively, of northeastern Sulawesi Island of Indonesia (Aoyama et al. 2003). These studies indicate that in contrast to the long migrations made by temperate eels, tropical eels make much shorter migrations to spawn in areas near to their freshwater habitats. However, the natural reproductive ecology and spawning patterns of tropical eels as well as temperate eels have remained a mystery, and it has thus remained difficult to determine the nature of the migrations of freshwater eels.

The aim of this study was to examine the nature of primitive forms of spawning ecology and migration mechanisms in freshwater eels using spawning-condition tropical eels. The spawning-condition tropical eels *Anguilla celebesensis* and *A. marmorata* were collected in 2008 and 2009 from Lake Poso, central Sulawesi, Indonesia, which is connected to Tomini Bay by the Poso River. The gonadal histology of the tropical eels also showed mid-vitellogenic oocytes in final preparation for spawning. These findings suggest that freshwater eels originally would migrate only short distances to local spawning areas. Following the passive, long-range dispersion of their leptocephalus stages by currents, freshwater eels evolved long-distance migrations to return from their temperate growth habitats to their tropical spawning grounds.

## Material and Methods

**Figure 1:**
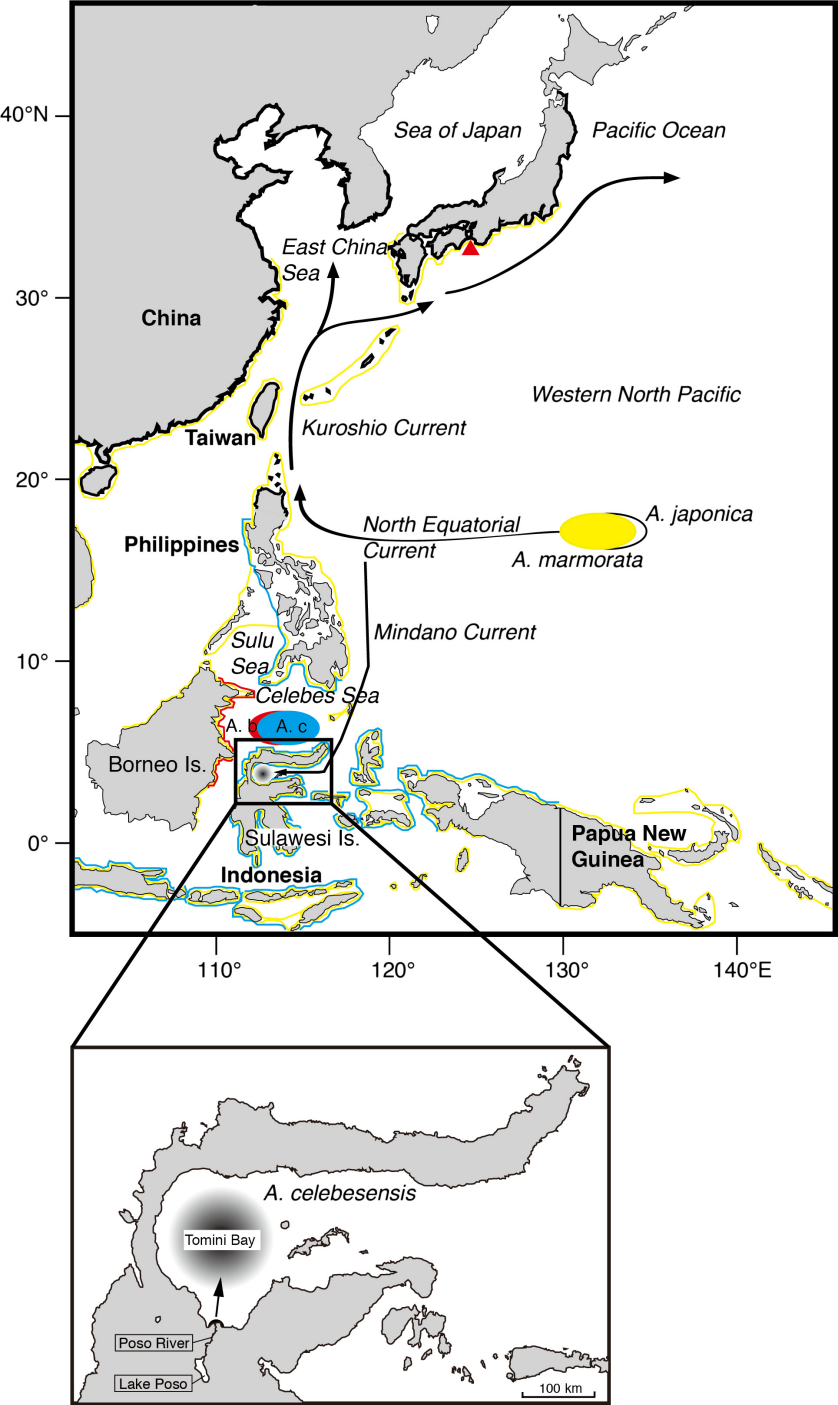
Map of the locations where the tropical eels *Anguilla celebesensis* and *A. marmorata* were collected, a possible spawning area of *A. celebesensis* in Tomini Bay (Aoyama et al. 2003), central Sulawesi of Indonesia and the short-distance migratory route of *A. celebesensis*. The spawning areas of *A. celebesensis* and *A. borneensis* found in the Celebes Sea (Jespersen 1942; Aoyama et al. 2003) are shown with their general distribution ranges (blue lines on coastlines in *A. celebesensis* and red lines on coastlines in *A. borneensis*). The locations of the offshore spawning areas with the migratory routes and the distribution ranges of *A. marmorata* (yellow lines on coastlines) and *A. japonica* (black lines on coastlines) are also illustrated to compare the local short-distance spawning migrations of *A. celebesensis* and *A. borneensis* with those of long-distance spawning migration species. A. c: spawning area of *A. celebesensis*, A. b: spawning area of *A. borneensis*, red triangle: sampling site of *A. japonica* specimens.

A total of 41 *Anguilla celebesensis* and 64 *A. marmorata* were collected inlet and outlet of Lake Poso and 13 *A. marmorata* were collected by eel traps from the mouth of Poso River, central Sulawesi Island, Indonesia between February 2008 and July 2009 (Fig.1). The lake is connected to the sea (Tomini Bay) by a single river, the Poso River, which drains the water from an elevation of 512 m past a waterfall and down to the sea along a 40-km stretch. To comparing the degree of maturation of temperate species during downstream migration season for spawning, a total of 37 migrating Japanese eel, *A. japonica*, were collected by bottom trawling in the Kii Channel off the eastern part of Shikoku Island, central Japan between December and January 2008 (Fig.1).

The whole gonad weight was measured and the gonadosomatic index (GSI; percent of the relative gonad weight to the body weight) was calculated. Fragments from the middle region of one gonad were fixed in formalin for histological analysis. Each maturation stage was modified according to Todd (1974) and Lokman et al. (1998). Stage I: Primary germ cells, oogonia, lamellae, and primary oocyte development were present at the early stage of oogenesis. Stage II: Development of immature cells with strongly basophilic cytoplasm and hematoxylin-eosin staining of primary oocytes with few oil droplets were observed. Stage III: This early maturation stage showed oocytes with cortical alveoli and oil droplets in their cytoplasm. Stage IV: Vitellogenic oocytes, nucleus, yolk granules, and central yolk platelets were observed. Oocyte diameter increased drastically and cytoplasm was filled with yolk granules. Stage V: Mid-vitellogenic oocytes in late preparation were observed for spawning. This stage involved nucleus, peripheral yolk granules, zona radiata, and vacuolated cytoplasm. Disintegrated nuclei were observed and whole nuclei were no longer visible. Zona radiata appeared as distinct striations took apart from granular follicular cells during late development.

The GSI values were compared among *Anguilla. celebesensis*,*A. marmorata* and *A. japonica*using the Kruskal–Wallis test. The significance of the correlation coefficient and the regression slope were determined using the t-test.

## Results

All of the *Anguilla celebesensis* specimens were females and the *A. marmorata* specimens were predominantly females (70%, *n* = 45), with only 10% (*n* = 6) of the total catch of males, and 20% (*n* = 13) was undifferentiated sex because the gonads did not develop. The Japanese eels that were collected offshore were predominantly females (89%, *n* = 33), with only 11% (*n* = 4) of the total catch of males. I used only female data for further analysis.

**Figure 2:**
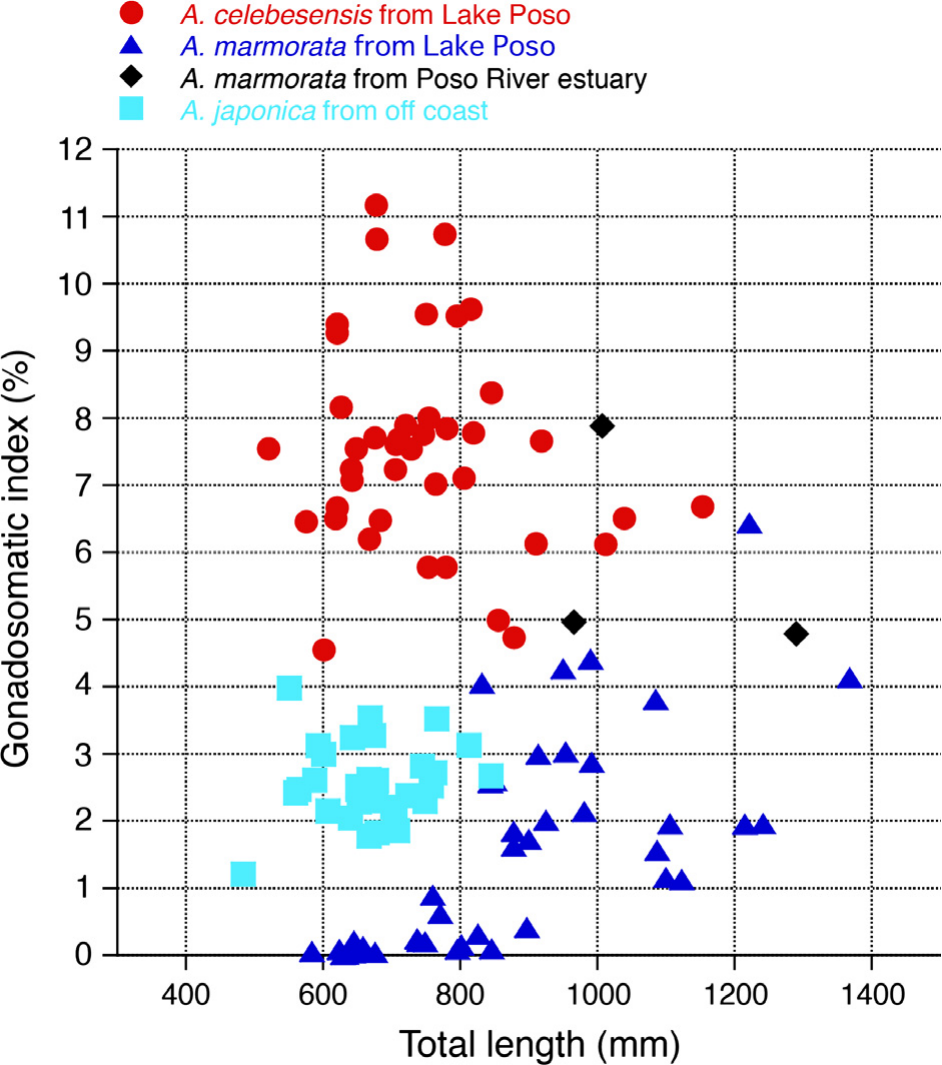
The GSI values of the tropical eels *Anguilla celebesensis* and *A. marmorata* that were collected from Lake Poso and an estuary of the Poso River in 2008 and 2009, and migrating silver eels of *Anguilla japonica* that were collected off the Shikoku Island of central Japan in 2008.

**Figure 3:**
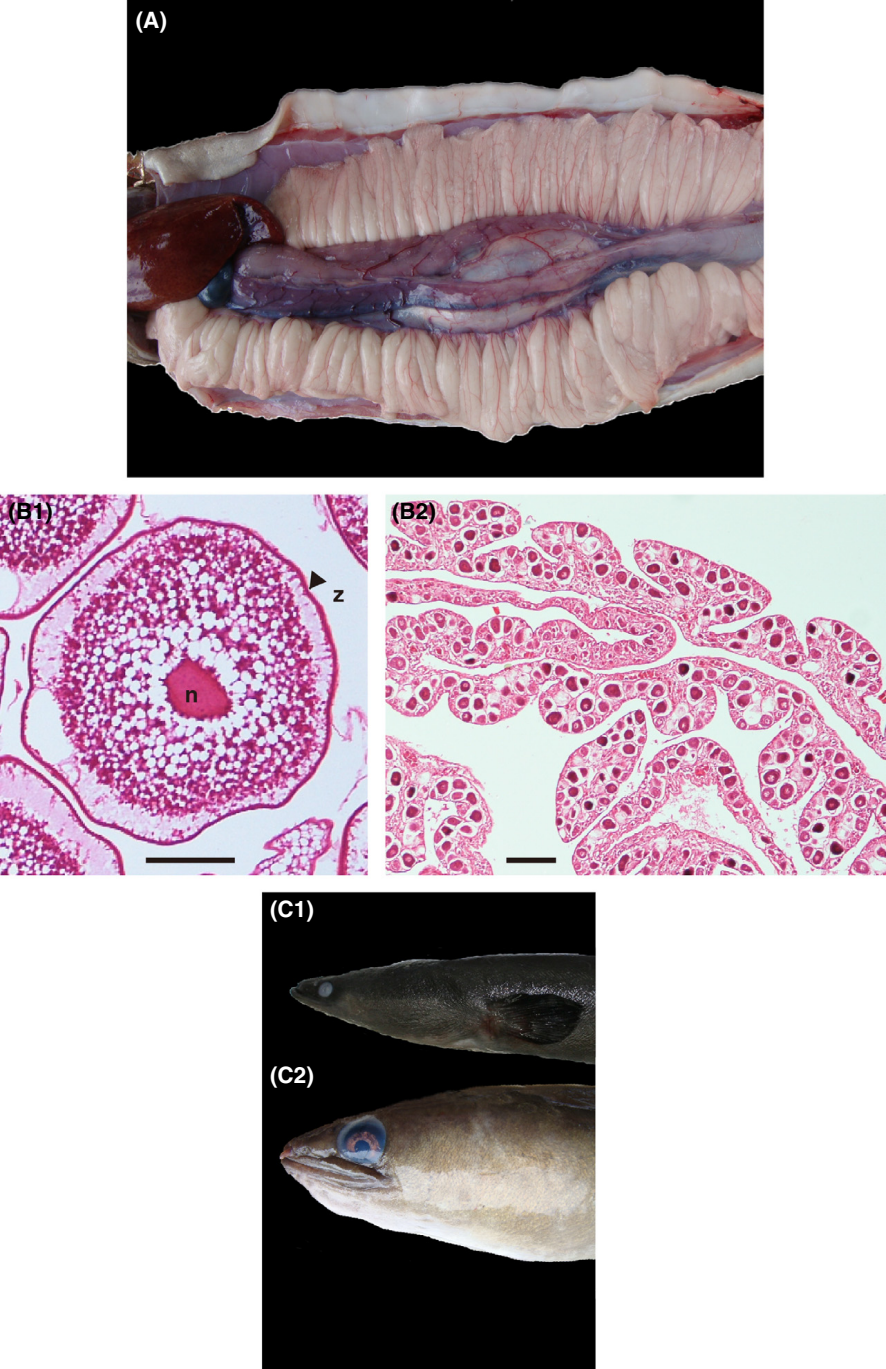
Gonadal (A) and histological (B-1) morphologies of spawning-condition tropical freshwater eels *Anguilla celebesensis* (754 mm in TL) that were collected in February 2009. The eye morphologies of migrating silver *A. japonica* eels that were collected off the Shikoku Island of central Japan in 2008 (C-1) and spawning-condition *A. celebesensis* that were collected from Lake Poso in February 2009 (C-2). In the spawning-condition tropical freshwater eels, the gonad was fully developed for spawning and the enlargement of eyes was greater (B-1). The gonadal histology of Stage V (B-1) showed mid-vitellogenic oocytes in the final preparation for spawning. In the early maturation stage of Stage I showed primary germ cells, oogonia, lamellae, and primary oocytes development were present at early stage of oogenesis in *A. marmorata* (B-2). n: nucleus, z: zona radiate, scale bar: 100 *μ*m.

The GSI of *Anguilla celebesensis*,*A. marmorata* and *A. japonica* ranged from 4.6 to 11.2, from 0.0 to 6.4, and from 1.2 to 4.0, respectively (Fig.2). The GSI values >1.0 were classified as migrating silver (maturing) stage in *A. japonica*; therefore, all of the *A. japonica* specimens from the coastal area were silver eels in this study. The GSI values of *A. celebesensis* were significantly greater than those of the migrating silver-stage *A. japonica* and *A. marmorata* (*P* < 0.0001). All of the *A. celebesensis* specimens had greater GSI values than those of the migrating silver-stage *Anguilla japonica* specimens from the coastal areas. Eight *A. marmorata* (mean ± SD: 1078 ± 190 mm, range 832 to 1368 mm in total length) showed GSI values >4.0, which was the maximum value of *A. japonica* in the silver stage. The gonads were well developed morphologically (Fig.3A). These eels were silvery dark brown in color with greatly enlarged eyes (Fig.3C-1, C-2).

**Figure 4:**
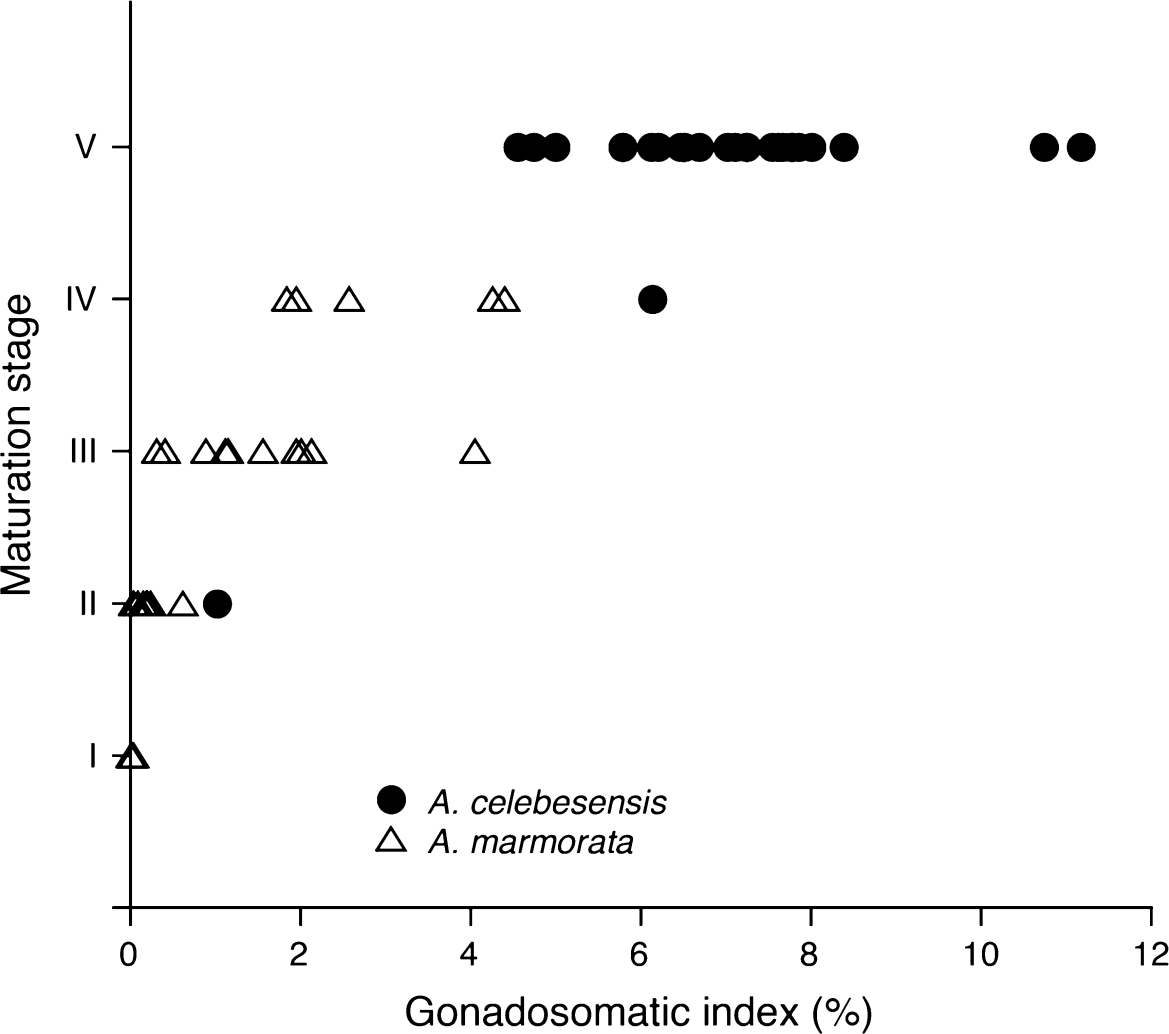
Relationship between gonadosomatic index and maturation stage for the tropical eels *Anguilla celebesensis* and *A. marmorata* that were collected from Lake Poso and an estuary of the Poso River in 2008 and 2009.

The gonadal histology of all of the *A. celebesensis* specimens had GSI values >9.0, indicating mid-vitellogenic oocytes in final preparation for spawning (Fig.3B-1). Compared with the postspawning stage, many postovulated follicles accumulated peripheral tissue (Fig.3B-1). However, mid-vitellogenic oocytes were present suggests that the eels were in the final growth stage of active spawning. In *A. celebesensis* and *A. marmorata*, significant positive correlations were found between maturation stage and GSI values (Fig.4) (*P* < 0.0001). Almost all *A. celebesensis* specimens showed the most advanced stage of Stage V, while the developmental stage varied in *A. marmorata* from Stage I (Figs.3B-2) to Stage V (Fig.4).

## Discussion

The findings reported here indicate that freshwater eels living in tropical regions would have life history characteristics that differ markedly from those of their temperate relatives, which have a single spawning site for each species, long-spawning migrations in both the North Atlantic (Schmidt 1922) and North Pacific Ocean (Tsukamoto 1992), and a distinct spawning season. Temperate anguillids have clear seasonal patterns of downstream migration, spawning in the open ocean, and recruit of glass eels (Tesch 2003). However, the present study clearly demonstrated that the spawning area of a tropical eel, *Anguilla celebesensis*, is a short distance from their freshwater habitat. A few small leptocephali of *A. celebesensis* (12–20 mm) and *A. borneensis* (8–13 mm) were found in the Celebes Sea (Jespersen 1942; Aoyama et al. 2003) where is also adjacent to their growth habitats (Fig.1). These suggest that those two species spawned in the area and the spawning migration in *A. borneensis* would be also quite short distance. Interestingly, spawning areas of *A. celebesensis* have been found at least two sites, that is, the Tomini Bay and the Celebes Sea, around Sulawesi Island, which is in clear contrast to their temperate counterparts (Fig.1).

Migrating silver eels of a few species have been caught incidentally along continental margins in both the Atlantic and Pacific oceans (Wenner 1973; Ernst 1977; Bast and Klinkhardt 1988; Sasai et al. 2001; Kotake et al. 2005; Chino and Arai 2009) but never close to their spawning areas until recently. Downstream migrating female European eels typically have GSI values >1.2 (Vøllestad and Jonsson 1986; Durif et al. 2005) but <3.0 (Svedäng and Wickström 1997; Durif et al. 2005). The levels of female maturation (GSI) of Japanese eels as they began their spawning migration in coastal areas ranged from 1.0 to 4.0 similar to that of eels that were collected in the East China Sea and other Japanese coastal areas (Sasai et al. 2001; Kotake et al. 2007). In this study, the GSI values of all of the *Anguilla celebesensis* specimens were >4.0. Furthermore, the present study found spawning-condition *A. celebesensis* with considerably high GSI values of >9.0 in the uppermost lake in central Sulawesi. The gonadal histology of the eels also indicated that the eels were in the final preparation for spawning. The eel larvae have higher tolerance and adaptability to lower salinity environments (Okamura et al. 2009). The hatched eel larvae (7 to 9 days old) were adaptable to 5–20 psu brackish water, and the best survival rate was recorded at 10 psu (Chang et al. 2004). These results indicate that freshwater eels have a condition that is able to spawn in river downstream, although spawning of freshwater eels have never been discovered in river and estuary.

Before beginning their oceanic migrations, freshwater eels metamorphose from yellow (immature) to silver (mature) in fresh water and estuarine habitats. This process begins weeks to months before migration (Fontaine et al. 1995). In temperate eels, the process of maturation is not completed during the early stages of the migration out of freshwater, estuarine, and coastal habitats because the GSI values are still quite low, and the gonads are also not fully developed histologically, indicating that the process of gonadal maturation mostly occurs during their thousands of km oceanic spawning migrations of silver eels and when the eels reach the offshore spawning area. However, the spawning area of *Anguilla celebesensis* is thought to be located in Tomini Bay adjacent to their growth habitats (Fig.1, Aoyama et al. 2003). The Poso River flows into the southern part of the bay and is the largest drainage and potential source of silver eels adjacent to the bay (Fig.1). Such a considerably short spawning migration of <100 km may induce the final stage of maturation in inland water for a short period time to reach the spawning area.

**Figure 5:**
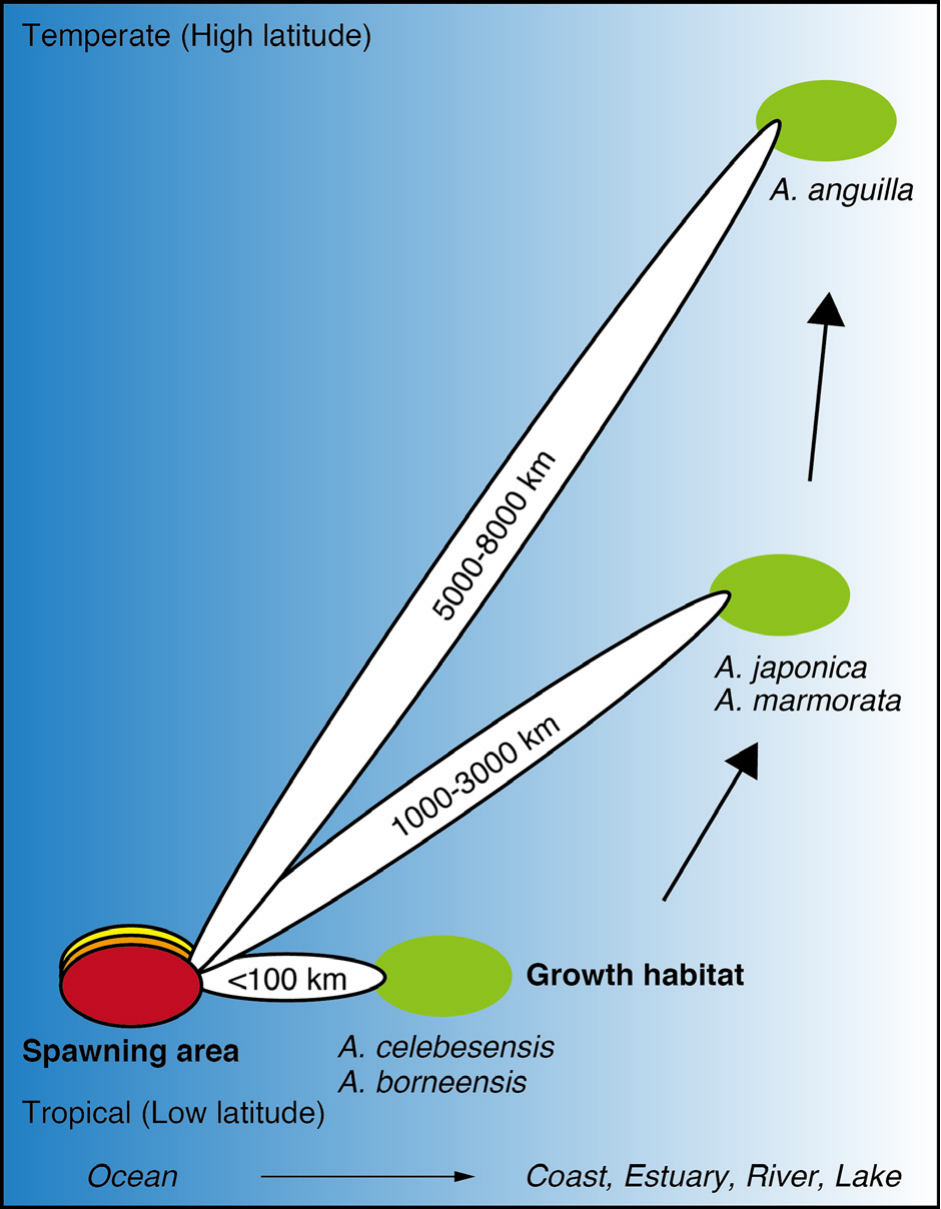
Scenario of the expansion of catadromous eel migration in ancestral eels from a local short-distance movement in tropical regions to a long-distance migration in temperate regions.

The GSI values of *Anguilla marmorata* from both the Lake Poso and the River Poso were greater than those of temperate silver eels, and the several eels had levels that were high but less than those of *A. celebesensis* (Fig.2). The spawning area of *A. marmorata* in the Poso population may be located far from Tomini Bay. Recently, one spawning-condition female *A. marmorata* was collected in the western North Pacific where is the spawning ground of the Japanese eel *A. japonica* (Tsukamoto et al. 2011; Fig.1) corresponded to the estimated spawning area in *A. marmorata* by Arai et al. (2002) and Kuroki et al. (2006) as determined by otolith analyses. Thus, the western North Pacific is a possible spawning area in *A. marmorata* from central Sulawesi belonging to the North Pacific population (Minegishi et al. 2005) (Fig.1). The spawning migration of *A. marmorata* ranges from 1000 km to 3000 km being similar to that of *A. japonica* (Figs1 and 5), and it was quite different from those of *A. celebesensis* and *A. borneensis* (Figs1 and 5). *A. marmorata* is a unique tropical anguillid that reaches large sizes of almost 2 m in length with a maximum weight of 21 kg (Castle 1984). This species has the widest geographic distribution from temperate to tropical regions in the 19 species of freshwater eels of the genus *Anguilla* (Ege 1939) and is found longitudinally from the east coast of Africa to the Marquesas Islands in the southeast Pacific Ocean and as far north as southern Japan (Ege 1939). Recently, this species was found at the Palmyra Atoll in the central Pacific (Handler and James 2006) and even farther to the east in the Galapagos Islands (McCosker et al. 2003), which may indicate that it has an even wider geographic range than previously thought. These results suggest that larger larval dispersal in ocean and higher adaptation for various ambient environment after recruitment to growth habitats might be able to have the long-distance spawning migration in *A. marmorata*.

The spawning areas of freshwater eels are all located in the southern regions. Analyses of the otolith microstructure showed that the ages at recruitment of tropical eels were constant throughout the year (Arai et al. 2001). The spawning seasons of tropical eels were found to extend throughout the year (Arai et al. 2001). The year-round spawning of tropical species and constant larval growth throughout the year extend the period of recruitment to estuarine habitats to year-round in tropical eels (Arai et al. 1999; Sugeha et al. 2001). Local short-distant migration made by tropical eels enables such spawning ecology and recruitment mechanisms. For temperate eels, the retention of their spawning areas in the tropics would require that the eels migrate thousands of kilometers to have clearly seasonal patterns of downstream migration, spawning in the open ocean, and recruitment of glass eels.

Ancestral eels most likely underwent a catadromous migration from a local, short-distance movement in tropical coastal waters using simple migratory mechanisms, subsequently developing the long-distance migration characteristic of present-day temperate eels, well established as occurring in a subtropical gyre in both hemispheres (Fig.5).
